# A Novel Simulation Technician Laboratory Design: Results of a Survey-Based Study

**DOI:** 10.7759/cureus.534

**Published:** 2016-03-16

**Authors:** Rami Ahmed, Patrick G Hughes, Ed Friedl, Fabiana Ortiz Figueroa, Jose R Cepeda Brito, Jennifer Frey, Lauren E Birmingham, Steven Scott Atkinson

**Affiliations:** 1 Emergency Medicine, Summa Akron City Hospital, Summa Health System; 2 Construction & Property Management, Summa Akron City Hospital, Summa Health System; 3 Performance Solutions Department, Summa Akron City Hospital, Summa Health System; 4 Medical Education, Summa Akron City Hospital, Summa Health System

**Keywords:** simulation laboratory design, simulation technician, simulation specialist training

## Abstract

**Objective:**

The purpose of this study was to elicit feedback from simulation technicians prior to developing the first simulation technician-specific simulation laboratory in Akron, OH.

**Background:**

Simulation technicians serve a vital role in simulation centers within hospitals/health centers around the world. The first simulation technician degree program in the US has been approved in Akron, OH. To satisfy the requirements of this program and to meet the needs of this special audience of learners, a customized simulation lab is essential.

**Method:**

A web-based survey was circulated to simulation technicians prior to completion of the lab for the new program. The survey consisted of questions aimed at identifying structural and functional design elements of a novel simulation center for the training of simulation technicians. Quantitative methods were utilized to analyze data.

**Results:**

Over 90% of technicians (n=65) think that a lab designed explicitly for the training of technicians is novel and beneficial. Approximately 75% of respondents think that the space provided appropriate audiovisual (AV) infrastructure and space to evaluate the ability of technicians to be independent. The respondents think that the lab needed more storage space, visualization space for a large number of students, and more space in the technical/repair area.

**Conclusions:**

A space designed for the training of simulation technicians was considered to be beneficial. This laboratory requires distinct space for technical repair, adequate bench space for the maintenance and repair of simulators, an appropriate AV infrastructure, and space to evaluate the ability of technicians to be independent.

## Introduction

Medical simulation centers are now commonly found in medical schools and teaching hospitals throughout the United States [1]. Simulation technicians serve a vital role within these facilities [2]. Simulation technicians must have a broad knowledge base in pathophysiology, pharmacology, simulation scenario design, simulator setup/maintenance, and administrative training [3]. The simulation technician role is still loosely defined. A recent survey from Cincinnati Children's Hospital Medical Center showed that simulation technicians identified five core tasks as their primary roles: equipment setup and breakdown, programming scenarios into software, operation of software during simulations, audiovisual (AV) support, and on-site simulation maintenance [4]. The same survey also noted that simulation technicians thought they lacked formal training and they bridged the self-perceived educational gap by self-directed learning [4].

A majority of simulation technicians feel unprepared for their role upon starting their careers [4]. Driven by this identified educational need, a comprehensive simulation technician curriculum was designed as a two-year associate degree program and has been approved by the Ohio Department of Higher Education at the University of Akron in collaboration with Summa Health System in Akron, Ohio. This program is the first of its kind in the United States [5]. A customized simulation lab for these learners was deemed necessary to meet the unique educational needs of this novel curriculum. This space requires a dedicated area for simulation technician students that provides hands-on experiences to learn simulator repair, AV system setup, as well as to run simulations under faculty supervision. The objective of this study was to obtain feedback from simulation technicians around the world prior to breaking ground on this novel space and to scrutinize our originally planned lab design and layout.

## Materials and methods

### Survey instrument

**Figure 1:**
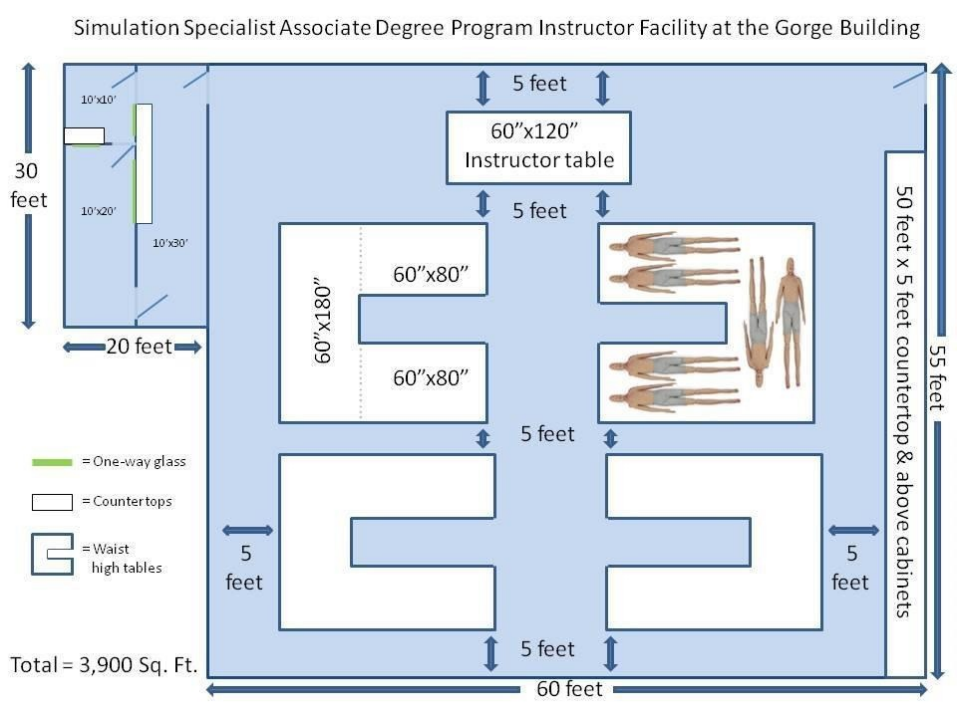
Image of blueprint

A structured survey was developed by the research team and disseminated with Survey Monkey (www.surveymonkey.com). The survey presented a blueprint of a simulation lab and then asked the participants to rate the adequacy of the space on several criteria using a five-point Likert-style scale (e.g., adequacy of student working space, adequacy of audiovisual infrastructure, storage space, etc.) (Figure 1). Demographic information was also gathered, and space was provided for the participants to write more detailed thoughts about the design of the simulation lab space. The study was considered exempt by the Institutional Review Board as it did not meet the definition of human subject research.

### Study subjects

This survey targeted simulation technicians. Email addresses were gathered from an email list of simulation specialists maintained by a professional organization. The participants were recruited through an email invitation to take an online survey about the adequacy of a customized training space for simulation technician training. An email invitation was sent to all 265 members on the list. The online survey was available from August to September 2015. Three reminder emails were sent during this time.

### Capabilities of the space: technical report

**Figure 2:**
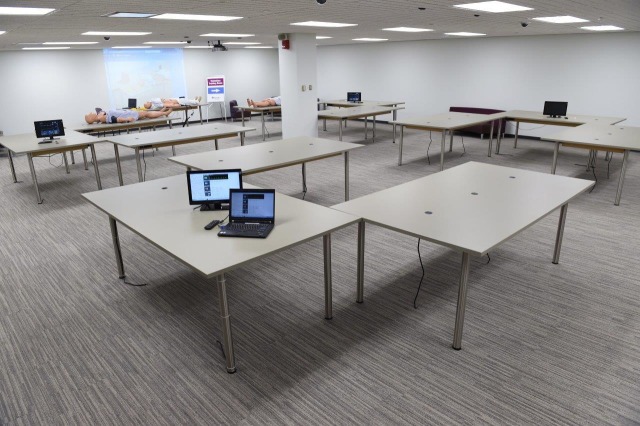
Technician classroom

The classroom and technical repair laboratory facilitates the development of skilled technicians capable of troubleshooting problems with the simulator's internal hardware, software, and mechanics. Trained technicians will be capable of real time, on-site repairs, thus reducing down-time of simulators and costs by decreasing the need to return simulators to manufacturers for repairs. The large open concept classroom has enough table-top space to lay two full body simulators side-by-side on each station (three tables at each station). The table-top on these stations provides appropriate work space to allow students to work directly across from each other while visualizing and repairing a mannequin’s internal electronics (Figure 2). With the students sitting on the outside area of the U-shaped table, the Master Instructor can observe the students working on their own simulators and instruct multiple students simultaneously by using the inside area of the U-shaped table. This potentially provides an environment to learn about various types/manufacturers of simulators simultaneously. The open space design provides for effective, hands-on summative testing of technicians, allowing for station rotation, problem identification using diagnostic algorithms, followed by real-time repair, with multiple technicians being tested simultaneously.

The addition of an online audio/visual learning management system (LMS) permits the Master Instructor to see the students’ progress through a monitor located at the Master Instructor worktable. The monitor allows the Master Instructor to visualize all 24 potential students at once with the option to focus in on a single student with a mouse click. Additionally, the audio/visual LMS also allows each student to get a close-up view of the Master Instructor’s worktable, which provides detailed technical instruction and the ability to see the Master Instructor’s hands as he/she demonstrates the steps of a technical skill. This can be visualized through individual monitors placed at each student’s workstation. This configuration conveniently allows all students to follow along during class without needing to walk to each work table within the instructional facility or to be crowded around the Master Instructor's worktable. The facility also has a large liquid crystal display (LCD) 10 x12 foot screen located in the front and sides of the room. This screen offers a large audio/visual image for anyone not viewing their individual monitors. 

**Figure 3:**
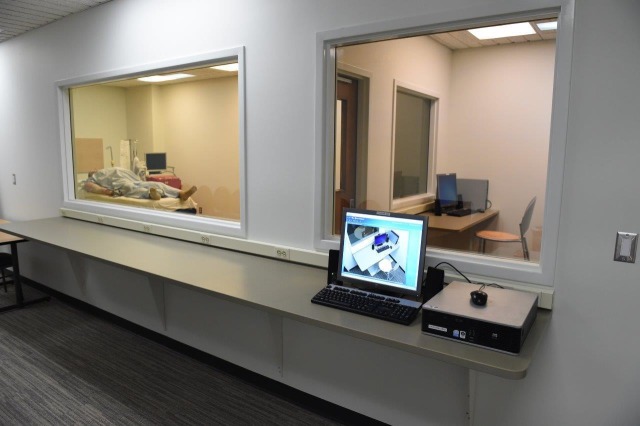
Technician control room training area ("Control room looking into a control room")

**Figure 4:**
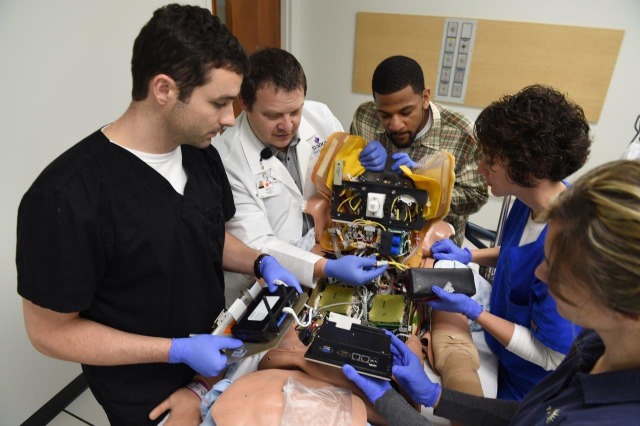
Technical repair training for simulation technician students

The ability to modify the simulator's vital signs based on the learner's decision-making during a simulation is an essential skill for the technician. However, there is more skill and preparation required for the technician to run a successful simulation. The clinical simulation lab provides an isolated environment to run a full simulation, manage the setup and breakdown of a simulation, and test, run, and troubleshoot the audiovisual components of a simulation. Once the technician is ready for supervised practice or is being tested to determine competency, the Master Instructor will be able to monitor the student’s progress by direct observation, from outside the control room. This is possible by means of direct observation through a one-way mirror from the instructor observation area (Figure 3). The instructor observation area sits in front of a large one-way glass that looks into the control room (“control room looking into a control room”) as well as the simulated clinical space that the technician will be working in. This environment provides the technician-in-training the opportunity to perform with complete autonomy in order to demonstrate their skill set and problem-solving strategies without prompting or assistance from instructors. This distinctive training space allows the Master Instructor to cut power, disconnect Wi-Fi and LAN lines, as well as communicate with standardized confederate actors portraying the role of learners, to create situations that the technician-in-training may not anticipate and will need to troubleshoot (Figure 4).

## Results

**Figure 5:**
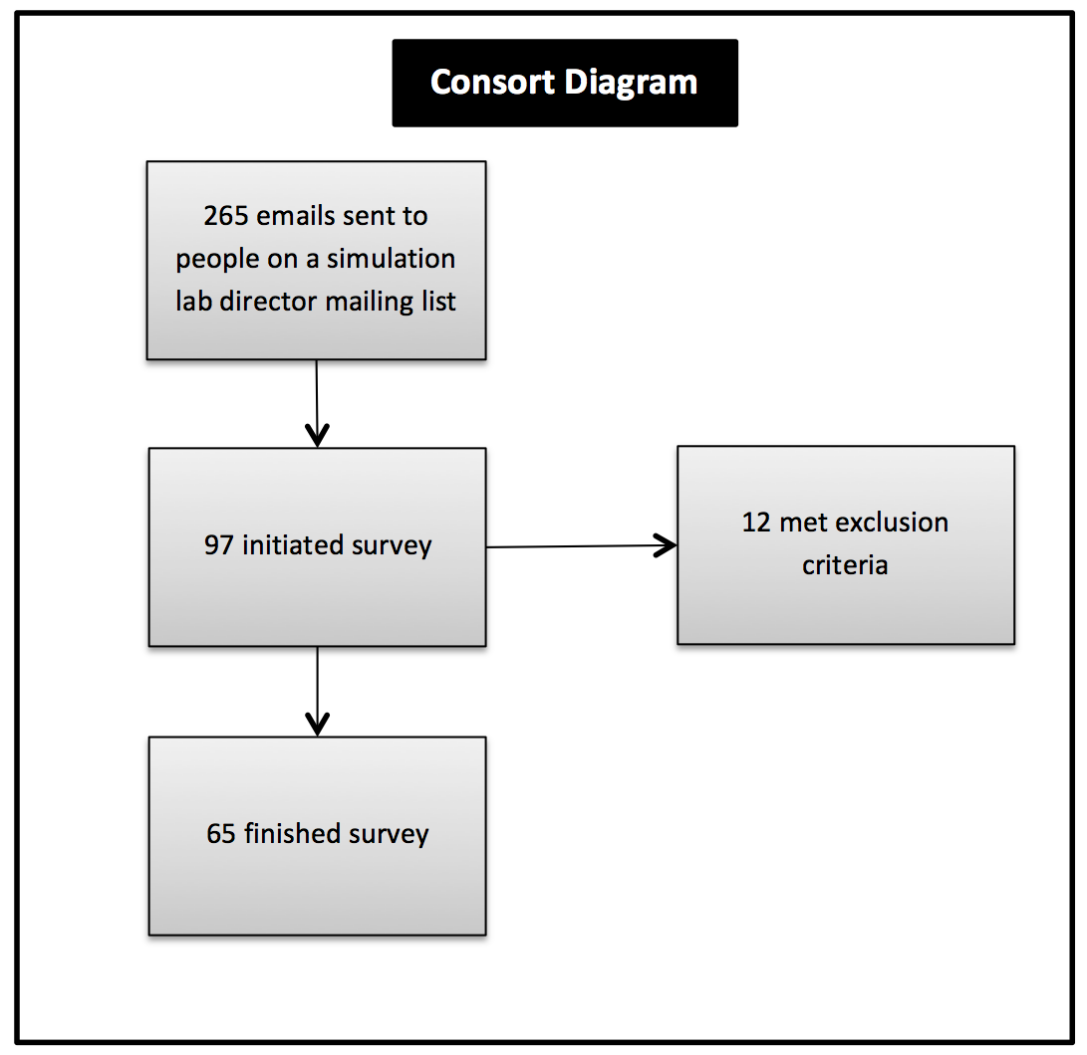
Consort diagram

Of the 265 individuals contacted for survey participation, 37% initiated the survey (n=98). There were two survey eligibility questions (age over 18 years and self-identified as a simulation specialist) which led to 12 participants being excluded. Twenty-five percent of individuals completed the survey (n=65) (Figure 5).

The survey participants represented four countries (United States, Canada, Mexico, and Germany). Most survey participants were male (57%, 34/60), non-Hispanic (93%, 53/57), and white (89%, 50/56). The mean years of experience as a simulation specialist was 7.4 ± 5.2 years. Most survey participants (72%, 42/58) had at least a bachelor's degree (bachelor's degree 43%, 21/58; master's degree 33%, 16/58; doctoral or medical degree (9%, 5/58).

Over 90% (59/65) of technicians agreed that a lab designed explicitly for the training of technicians is novel and beneficial. Approximately 75% of respondents indicated the space designed provided appropriate AV infrastructure (46/62) and space to evaluate the ability of technicians to be independent (50/66). The respondents thought the lab needed more storage space, visualization space for a large number of students, and more space in the technical/repair area for technicians (Table 1).

**Table 1:** Survey results

Opinions of Teaching Space	Strongly Disagree/ Agree	Neutral	Agree/ Strongly Agree
Classroom technical/repair area provides adequate working space	26 (38%)	6 (9%)	36 (53%)
Classroom technical/repair area provides appropriate area for formal testing	19 (28%)	7 (10%)	41 (61%)
Simulation technician training lab design/layout provides an environment that facilitates the development of technical skills	10 (15%)	11 (16%)	46 (69%)
Simulation technician training lab "control room looking into a control room" environment provides an appropriate space to evaluate the ability of the technicians to be independent	10 (15%)	6 (9%)	50 (76%)
AV infrastructure (LMS, overhead projector, etc.) provides suitable teaching instruments for student learning	5 (8%)	11 (17%)	47 (75%)
Classroom technical/repair area customized lab benches for training of software/hardware of simulators is adequate	12 (18%)	12 (18%)	41 (63%)
Side bench area is adequate	9 (14%)	18 (28%)	37 (58%)
Classroom observation area is adequate to visualize a simulation by a large number of students	23 (35%)	17 (18%)	31 (47%)
Teaching and training lab overall provides adequate storage	24 (38%)	17 (27%)	23 (36%)
I find the opportunity to utilize a unique environment customized for the development of technicians novel and beneficial	2 (3%)	4 (6%)	60 (91%)

## Discussion

This lab was designed to facilitate the training of expert technicians capable of a wide array of skills necessary to run and operate a simulation lab. As the utilization of medical simulation becomes more main-stream and the incorporation of simulation labs within hospitals, medical centers, and universities continues to expand, the formal training of personnel responsible for operations and maintenance of these labs is essential. Labs designed specifically for the training of technicians could ultimately be used to train and test faculty, fellows, or operations managers in many of the same skills in a train-the-trainer environment.

The data reveals that technicians think this space is a novel and cutting-edge environment for training. The audio/visual system combined with the large workstations provides an effective training environment for the development of master technicians. These large flat open U-shaped workstations allow students to work directly across from each other and visualize/repair the mannequin’s internal electronics. With the students on the outside of the U-shaped workspace, the Master Instructor can enter inside the U-shaped space and instruct multiple students simultaneously.

Many respondents felt the viewing area into the clinical simulation classroom required a larger space. This design had space limitations due to its location and was retrofit into an existing area. Ideally, additional space would have been allocated to allow for approximately 24 students to directly visualize the clinical simulation classroom. A large screen TV will be added to the room for visualization from the LMS to help offset the lack of direct visualization due to the space constraints. Additionally, the LMS can be seen from afar in a large classroom or auditorium with no maximum viewing limit. 

As in many institutions, storage space was overlooked and insufficient. The majority of respondents felt that storage space could be increased. With our limited space allocation, we were unable to institute the industry standard of dedicating 20% of the available square footage for simulation equipment storage. To compensate for our storage deficit, we included storage cabinets along the main wall for student supplies. We also installed metal racking in the two control rooms to create additional storage space.

While the customized lab benches in the classroom were considered adequate by a majority of respondents, approximately 40% of respondents felt the classroom technical/repair area did not provide adequate working space. Our interpretation of these results led us to believe our aisleways for the space in-between the U-shaped work stations needs to be larger for transporting mannequins to the work stations. Institutions planning on developing such training facilities should account for appropriate space to transport large simulators, medical beds, crash carts, and other large equipment necessary for the repair and maintenance of such simulators between stations and storage.

This study had several limitations. The respondents were not given three-dimensional renderings of the blueprint, potentially limiting their ability to fully understand the layout of the space. A flat one-dimensional schematic was presented in the survey. This survey was anonymous and therefore demographics of those who did not complete the survey could not be compared to survey participants. Further research should investigate the perceptions of simulation technician graduates as it pertains to their curricula and how the utilization of the space met their educational needs.

## Conclusions

A space designed explicitly for the training of simulation technicians was considered beneficial by a significant majority of respondents. This laboratory requires distinct space for technical repair, adequate bench space for the maintenance and repair of simulators, an AV infrastructure that provides easy visualization and access to both students and instructors, and space to evaluate the ability of technicians to be independent.
